# A reduction in positive self-judgment bias is uniquely related to the anhedonic symptoms of depression

**DOI:** 10.1016/j.brat.2009.01.016

**Published:** 2009-05

**Authors:** Barnaby D. Dunn, Iolanta Stefanovitch, Kate Buchan, Andrew D. Lawrence, Tim Dalgleish

**Affiliations:** a Medical Research Council Cognition and Brain Sciences Unit, 15 Chaucer Road, Cambridge CB2 7EF, UK; b Sub-Department of Clinical Health Psychology, University College London, Gower Street, London WC1E 6BT, UK; c Wales Institute of Cognitive Neuroscience, School of Psychology, Cardiff University, Tower Building, Park Place, Cardiff CF10 3AT, UK

**Keywords:** Depression, Anxiety, Tripartite model, Positive bias

## Abstract

Identifying patterns of biased cognitive processing specific to depression has proved difficult. The tripartite model of mood disorders [Clark, L. A., & Watson, D. (1991). Tripartite model of anxiety and depression: psychometric evidence and taxonomic implications. *Journal of Abnormal Psychology, 100*, 316–336] suggests that a clearer processing ‘blueprint’ may emerge if depression is viewed dimensionally rather than categorically and by focusing on variations in the degree of positive, rather than negative, processing bias. To investigate this possibility, the present study examined the extent to which a reduced positive self-judgment bias previously found in depressed individuals relates to depression-specific anhedonic symptoms. Sixty participants with varying levels of anxiety and depression symptoms evaluated their own performance on a working memory task in the absence of external feedback. Overall, participants showed a positive self-judgment bias, overestimating the number of trials they had performed correctly relative to objective criteria. Consistent with the tripartite framework, the extent of this positive self-judgment bias was significantly and uniquely related to depression-specific symptoms, with the positive bias reducing as anhedonia severity increased across three different symptom measures.

## Introduction

A central theme of contemporary emotion research is that biased cognitive processing is an intrinsic component of emotional disorders (Mineka, Rafaeli, & Yovel, 2003). Similarly, a core tenet of cognitive treatments for mood disorders is that identifying and normalising biased processing can ameliorate symptoms (Clark, Beck, & Alford, 1999). Considerable effort has therefore been made to identify any unique cognitive profiles associated with different forms of emotional disorder, both to aid differential diagnosis and to help with the development of more effective clinical interventions. This has particularly been the case for anxiety and depression (Williams, Watts, MacLeod, & Mathews, 1997), amongst the most common yet debilitating conditions seen in routine clinical practice.

This cognitive profiling addresses two different questions: first, whether depressed individuals are more likely to exhibit a bias for depression-relevant information, and anxious individuals for anxiety-related information, than vice versa (‘content’ specificity); second, whether depression is characterised by bias in particular cognitive processes that are not altered in anxiety, and vice versa (‘process’ specificity).

There is growing support for content-specificity, with depression being associated particularly with a focus on themes of loss and failure and anxiety leading to a preoccupation with psychological or physical threat (e.g. Clark, Beck, & Stewart, 1990; Ingram, Kendall, Smith, Donnell & Ronan, 1987; for a review see Beck, 2005). The case for process-specificity is less certain. Equivocal results have emerged from an extensive literature examining a range of cognitive processes in the affective disorders, including judgment biases (interpreting ambiguous information in a positive or negative direction) and attention and memory biases (showing preferential attention or recall of positive or negatively valenced material).

In the judgment domain, diagnoses of depression or anxiety have both been associated with negative interpretation of ambiguous information (e.g. Butler and Mathews, 1987; Eysenck, Mogg, May, Richards, & Mathews, 1991; Lawson, McLeod, & Hammond, 2002) but there is little solid evidence of disorder specificity (Mineka et al., 2003). In the attention and memory domain, anxiety disorders have been reliably associated with attentional biases (preferential automatic and/or strategic processing of threat related material), with an inconsistent picture emerging regarding memory biases. In depression the converse has generally been found, with reliable evidence for memory biases (preferential recall of negative material) but mixed evidence regarding attentional biases (Mineka et al., 2003; Williams et al., 1997). Given these inconsistent yet sometimes positive patterns of memory biases in anxious groups and attentional biases in depressed groups, it is difficult to argue that these information processing styles can fully and satisfactorily differentiate between diagnoses of anxiety and depression.

Perhaps the strongest way to investigate processing specificity at the diagnostic level is to examine ‘pure’ anxious and depressed individuals on the same task(s) within the same experiment. Only a relatively small subset of studies have adopted this approach, however (e.g. Bradley, Mogg, & Williams, 1995; Dalgleish et al., 2003; Dozois & Dobson, 2001; Dozois & Frewen, 2006; Heidenreich, Junghanns-Royack, & Stangier, 2007; Lim & Kim, 2005; Mathews, Ridgeway, & Williamson, 1996; Mogg, Bradley, & Williams, 1995; Mogg, Millar, & Bradley, 2000; Rinck & Becker, 2005) and even fewer have demonstrated clear processing specificity effects, not all of which are readily interpretable in the light of the previous literature. The relative dearth of these studies is likely to in part reflect the difficulty in recruiting ‘pure’ depressed and anxious populations.

In summary, experimental attempts to identify unique cognitive processing profiles that can distinguish depression and anxiety at the level of diagnosis have met with mixed results to date and there is a clear need for further research, to aid both theory and clinical practice. This is the aim of the present study.

These existing attempts to demonstrate processing specificity for anxiety and depression have almost invariably been underpinned by the current DSM diagnostic classification system (or sub-clinical analogues thereof), which presupposes that psychological distress can be classified into distinct types on the basis of criteria lists with unique defining features (e.g. “depressed”, “anxious”, or “healthy”). It is, however, increasingly a matter for debate whether such clear divisions exist between different emotional disorders. This is particularly the case for anxiety and depression, given the high levels of comorbidity between them, the tendency for individuals to move between diagnostic classes over relatively short time spans, and the frequent ‘boundary disputes’ that occur about how best to classify presentations that fall between categories (Mineka, Watson, & Clark, 1998; Widiger & Samuel, 2005).

Therefore, the case has been made that mental health complaints may be better conceptualised as at least partly dimensional rather than fully categorical constructs, with individuals falling somewhere on a series of approximately normally distributed and overlapping continua ranging from symptom free to highly symptomatic (e.g., Watson, 2005; Watson & Clark, 2006). Some of these symptom dimensions are relatively common across diagnoses, whereas others are relatively specific to a given diagnosis. For example, the influential tripartite framework (Clark & Watson, 1991; Clark, Watson, & Mineka, 1994) proposes that the symptoms of depression and anxiety are captured by three dimensions. Common to both conditions is ‘general distress’, consisting of non-specific ‘Negative Affect’ symptoms (e.g., depressed mood, anxious mood, insomnia). Unique to anxiety is physiological hyper-arousal and somatic tension (e.g., dizziness, shortness of breath), whereas unique to depression are ‘Positive Affect’ symptoms of anhedonia and apathy (e.g., loss of interest, feeling nothing is enjoyable). The Mood and Anxiety Symptom Questionnaire (MASQ; Watson & Clark, 1991; Watson, Weber, Assenheimer, Clark, Strauss, & McCormick, 1995) was developed as a self-report measure of these three symptom clusters and factor analytic studies have generally supported this conceptualisation (Mineka et al., 1998).^1^

It is perhaps unsurprising that reliably demonstrating processing bias specificity in anxiety and depression at the level of diagnosis has proved somewhat elusive, given the common ‘Negative Affect’ core the tripartite model postulates they share. Instead, it may be more fruitful to see if there are unique processing profiles associated with the putative depression-specific (anhedonia) and anxiety-specific (hyper-arousal) symptom dimensions. Surprisingly, this possibility has received relatively little research attention to date (for exceptions see Larson, Nitschke, & Davidson, 2007; Yovel & Mineka 2004, 2005).

The tripartite framework also raises another possible reason as to why previous attempts to identify cognitive distinctions between anxiety and depression at the categorical level have not been entirely successful. Existing studies have tended to focus predominantly on negative information processing biases, for example processing that favours negative (relative to neutral) cue words or negative evaluations of ambiguous events. However, if processing specificity in any way mirrors the specificity of symptom clusters, as outlined in the tripartite model, then such negative biases are likely to be common to both anxiety and depression. In contrast, a reduction in the extent of any *positive* bias may be uniquely associated with depression.

There is some support in the literature for an association between reduction in positive bias and depression assessed as a categorical diagnostic construct. While healthy individuals show a number of protective positive biases, for example over-estimating their degree of control and performance in a range of scenarios (Taylor & Brown, 1988; Mezulis, Abramson, Hyde, & Hankin, 2004), depressed individuals show a less positive (and more realistic) estimation of control (e.g. ‘depressive realism’; Alloy & Abramson, 1979; for a review see Haaga & Beck, 1995). Further, healthy individuals exhibit an attentional bias towards positive stimuli and/or away from negative stimuli, whereas depressed and dysphoric individuals exhibit ‘even-handed’ attentional deployment to positive and negative stimuli (e.g., Gotlib, McLachlan, & Katz, 1988; McCabe, Gotlib, & Martin, 2000; McCabe & Toman, 2000). Finally, while healthy individuals recall more positive than negative experimentally presented stimuli, depressed individuals recall similar numbers of positive and negative, or more negative than positive, stimuli (so-called ‘mood congruent recall effects’; for reviews see Blaney, 1986; Matt, Vázquez, & Campbell, 1992).

Surprisingly, the specificity of these blunted positive bias findings to depression, versus anxiety, has been investigated in relatively few studies to date and where such an approach has been adopted somewhat equivocal findings have emerged. Individuals with ‘pure’ or mixed depression were shown to endorse fewer positive adjectives as self descriptive, subsequently recall fewer of these positive items, and demonstrate less positive attribute redundancy, relative to ‘pure’ anxiety and control groups (Dozois & Dobson, 2001). However, there were no group differences for interference effects to positive words on the emotional Stroop task or for interconnectedness of positive interpersonal content in this study. Later investigations did find reduced interconnectedness for positive interpersonal content in depressed individuals, relative to healthy control participants and a mixed anxiety control group (Dozois & Frewen, 2006), although this profile did not show specificity to depression in that similar findings emerged for a sample with social phobia.

There is also some evidence that blunted positive future memory (i.e. a reduction in the number of anticipated positive events) and impaired recall of happy faces may be characteristic of mixed depression and anxiety but not pure anxiety (McLeod & Byrne, 1996; Gilboa-Schechtman, Erhard-Weiss, & Jeczemien, 2002). These studies did not however include a pure depressed group, weakening the support they can offer for the process specificity hypothesis.

In summary, these diagnostic studies have revealed promising, albeit equivocal, evidence for positive processing alterations being specific to depression. As detailed above, clearer support for attenuation of positive bias being specific to depression may be revealed by adopting a dimensional rather than diagnostic framework. However, as far as we are aware, no studies have yet taken this approach.

The present study is therefore an investigation of whether it is possible to separate depression more clearly from anxiety with respect to biased cognitive mechanisms when conceptualising the constructs in dimensional rather than categorical terms, and when focusing on variations in positive rather than in negative information processing biases.

To assess positive information processing biases we used a measure of self-regulatory judgment accuracy. A core presenting feature of mood disorders is the inability to regulate the self, where individuals report becoming overly internally preoccupied, experience unwanted negative affect, find it difficult to make decisions, and ruminate about their mood rather than responding adaptively to the demands of the environment (Gotlib & Hammen, 1992). These symptoms have been partly understood in terms of disturbances of a self-regulatory system (Carver & Scheier, 1998; Duval & Wicklund, 1972), whereby individuals iteratively evaluate the extent to which actual status matches ideal status on a self-relevant dimension using a ‘test-operate-test-exit’ (TOTE) function (Miller, Galanter, & Pribram, 1960). If current status falls short of the ideal goal individuals experience negative affect, motivating them to change their behaviour and then re-evaluate the consequences. At the point when the discrepancy between actual and ideal status is sufficiently small, the individual exits this cycle. In depression it is argued that individuals somehow become ‘locked’ in self-regulatory cycles, with unfavourable actual-ideal comparisons intensifying negative affect, lowering self-esteem, and impairing adaptive behaviour (Higgins, 1987; Ingram, 1990; Pyszczynski & Greenberg, 1987).

This difficulty in exiting self-regulatory cycles in depression may reflect an underlying bias in the way ideal-actual judgments are made (either showing an exaggerated negative or a reduced positive bias, relative to healthy individuals). To examine this issue we asked participants in a previous study to evaluate their own performance on a difficult spatial working memory task in the absence of any external feedback (B. D. Dunn, Dalgleish, Lawrence, & Ogilvie, 2007), therefore requiring them to make a self-regulatory judgment. Healthy control participants significantly over-estimated the proportion of trials they performed correctly, consistent with a positive judgment bias. Both dysphoric (Study 1: *n* = 20 per group) and clinically depressed (Study 2: *n* = 25 per group) individuals also displayed a positive judgment bias. However, the magnitude of this positive bias was significantly decreased, suggesting that depressed individuals may become locked in self-regulatory cycles because they are more sensitive in detecting a discrepancy between ideal and actual status.

For the purposes of the present study, this paradigm provides a robust measure of judgment bias, determining whether individuals overestimate (i.e. are positively biased), underestimate (i.e. are negatively biased), or are accurate about their performance in an ambiguous context where no feedback is provided. Importantly, the paradigm controls for a range of criticisms levelled against other positive judgment bias measures (e.g. contingency judgments; Haaga & Beck, 1995). First, bias is assessed relative to an objective performance baseline, making it possible to determine the absolute accuracy of judgments. Second, the same bias pattern is found in both dysphoric and clinically depressed populations, ruling out the possibility that the pattern of results is only found in mild depression and that a negative bias emerges in more severe cases. Third, task difficulty is titrated (increasing or decreasing the memory span depending on performance on the previous trial), thereby holding all participants at the upper ranges of their memory capacity. This means that altered judgment is not simply a consequence of the task being more effortful or demanding for depressed participants relative to control participants. This task therefore provides a sensible candidate measure to examine the extent to which the anxiety and depression specific dimensions of the tripartite model are related to variations in a positive judgment bias.

In the current study we employed a correlational design and administered this self-judgment task to participants presenting with a range of mixed mood disorder symptoms, from relatively symptom-free up to clinical levels. We examined the relationship between magnitude of positive bias and the symptom dimensions of the tripartite model (indexed using the short form of the MASQ [MASQ-S]; Watson & Clark, 1991). To further validate these results we performed similar analyses using anhedonia-related subscales of the Beck Depression Inventory (BDI-I; Beck, Ward, Mendelson, Mock, & Erbaugh, 1961) and the Spielberger State Trait Anxiety Inventory (STAI; Spielberger, Gorsuch, Lushene, Vagg, & Jacobs, 1983), two of the most widely used measures of dysphoric and anxious symptoms.

We had three hypotheses. First, based on literature arguing that healthy individuals display a range of protective ‘positive illusions’ (e.g. Taylor & Brown, 1988; Mezulis et al., 2004) and on our previous results (B. D. Dunn et al., 2007), we expected participants in general to show a positive bias on the task, over-estimating how well they were performing (Hypothesis 1). Next, we predicted that the extent of this positive distortion bias would decrease as anxious/depressed symptoms increased (Hypothesis 2). Finally, in line with the tripartite framework (Clark & Watson, 1991), we envisaged that symptoms specific to depression (anhedonia items from the MASQ, BDI-I and STAI) would account for this reduction in positive bias (Hypothesis 3). We addressed this in two ways. We initially examined whether the correlation between the anhedonia and positive bias was significantly stronger than the correlation between the remaining items and positive bias on all three scales. We then examined whether anhedonia remained significantly related to degree of positive bias, after partialling out scores on the remaining items on all three scales.^2^

## Method

### Participants

Sixty participants (44 women) aged 18–65 years (*M* = 43.98; *SD* = 15.00) with no history of brain injury, psychosis, learning disability or substance abuse were recruited (screened using a brief semi-structured interview enquiring about mental health and neurological history, current medications, and demographic characteristics). Individuals were selected from a database of community volunteers, which holds scores on dysphoria (BDI-I) and anxiety (STAI) measures from the previous occasion that individuals participated in a research study, therefore making it possible to ensure a broad range of anxiety and depression symptoms in the sample.^3^ Six participants were taking anti-depressant medication (five using SSRIs, one using a tricyclic). The mean estimated full scale IQ according to the National Adult Reading Test (NART; Nelson, 1982) was 116.15 (*SD* = 6.03). Participants gave written informed consent, were reimbursed the equivalent of US$10 per hour for their time, and the study was approved by the local research ethics committee.

### Self-judgment accuracy task

As in our previous study (B. D. Dunn et al., 2007), self-judgment was indexed in terms of participants’ accuracy in evaluating their own performance on a computerised spatial working memory task. On each of 20 trials, 8 red boxes were shown in fixed random positions on a black screen and a variable number of these boxes changed colour (to blue) in turn in a randomised order. After the boxes had finished changing colour, participants repeated the colour change sequence by clicking on each box in turn. At the end of each trial, they judged whether they had repeated the sequence correctly, in the absence of external feedback. The difficulty of the task was titrated to rule out performance confounds. Successful reproduction of a sequence led to an increase in the length of the sequence on the next trial, whereas incorrect reproduction led to a decrease (minimum sequence length = one, maximum sequence length = eight, first trial sequence length = five). This was designed to keep all participants at the upper end of their memory ability. This pattern was not explained to participants and none reported having noticed it when asked at the end of the experiment. Participants completed two practice trials to familiarise themselves with the task. Analysis focused on participants’ evaluation of their performance relative to objective criteria. This ‘distortion’ bias (from −1 to +1) was the proportion of trials estimated as performed correctly minus the proportion of trials actually performed correctly. A positive score therefore represents a positive distortion bias, participants judging that they performed more trials correctly than they actually did (for full task details, see B. D. Dunn et al., 2007).

### Symptom measures

The 62-item MASQ-S (Watson & Clark, 1991) was used to index depression-specific and anxiety-specific symptoms. Participants are asked to judge to what extent they have felt the way described in each question for the past week, ranging from one (not at all) to five (extremely). The MASQ-S comprises three factors. The general distress (GD) subscale has 11 items reflecting anxious mood (e.g. “felt nervous”) and 12 items reflecting depressed mood (e.g. “felt sad”), none of which strongly differentiates anxiety and depression (total score = 115). The anxious arousal (AA) subscale comprises 17 items assessing somatic tension and hyper-arousal (e.g. “was trembling or shaking”), which are believed to be relatively anxiety-specific (total score = 85). The anhedonic depression (AD) subscale has 8 questions assessing experiences believed to be specific to depression, including items measuring loss of pleasure and interest (e.g. “felt like nothing was very enjoyable”) and 14 reverse keyed items assessing positive emotional experience (e.g. “felt cheerful”) (total score = 110). The satisfactory reliability and validity of the MASQ-S has been well documented. In the present sample, internal reliability was acceptable for all three factors (Cronbach's *α* for AD = .94; for GD = .92; for AA = .83).

As additional measures of anxiety and dysphoria symptoms for examining Hypotheses Two and Three, participants completed the STAI state and trait scales (Spielberger et al., 1983) and the BDI-I (Beck et al., 1961). Previous studies suggest that these scales can also be sub-divided into anhedonic and non-anhedonic symptom clusters, even though this was not the primary intention when they were designed.

A factor analysis of the STAI trait scale (STAI-T) on a sample of 212 participants with mixed anxiety disorder diagnoses implemented by Bieling, Antony, and Swinson (1998) found a two dimension solution: a “depression” factor indexing symptoms related to blunted positive affect (items 1, 3–7, 10, 12, 16–19; Cronbach's *α* = .88) and an “anxiety” factor measuring symptoms of elevated negative affect (items 2, 8–9, 11, 13–15, 20; Cronbach's *α* = .78). For the purposes of the current study, the “depression” factor is used as a measure of anhedonia and is compared to the “anxiety” factor. Both factors displayed satisfactory internal consistency in the present sample (Cronbach's *α* = .92 for “depression” items and .85 for “anxiety” items).

We were unable to find published factor analytic studies of the STAI state scale (STAI-S) and therefore selected items measuring reduced “positive affect” identified by informal content analysis by Mathews and Mackintosh (2000) (items 2, 8, 11, 16, 20) as our measure of anhedonia and contrasted this to the remaining “other” STAI-S items. Internal consistency of these subscales in the present sample was also adequate (Cronbach's *α* = .80 for reduced “positive affect” items and .85 for remaining “other” items).

A factor analysis of the BDI-I conducted by Dunn et al. (2002) on 63 affective disorder patients (as part of a brain imaging study) revealed a five factor solution, including a “psychomotor anhedonia” factor (items 4,11–13 and 15). We contrasted this “psychomotor anhedonia” factor to the remaining “other” items in the present study. Cronbach's *α* for the “psychomotor anhedonia” and “other” factors (.79 and .83 respectively) again fell within acceptable limits in the current dataset.^4^

### Procedure

Participants were screened and the NART, STAI, BDI-I and MASQ-S were administered in turn. Volunteers then completed the self-judgment task, along with other measures not described here. Testing took place in a quiet, softly lit room, with participants seated in a comfortable chair facing the computer monitor.

## Results

For all analyses, alpha was set at .05. Statistical tests examining a priori hypotheses are directional and are noted as such in the text. Other tests are two-tailed. One participant's distortion score was an outlier and so was set aside from the analysis.

Participants completed approximately half of the sequences correctly on the self-judgment task (*M* = 0.52; *SD* = 0.04), indicating that task titration was successful, with a mean span length of 5.38 (*SD* = 0.60). There was no significant relationship between total scores on any of the mood measures and either mean memory sequence length or the proportion of sequences performed correctly, *P*s > .30, suggesting that mood disorder symptom severity did not influence memory performance.

As predicted in Hypothesis 1, most participants (92%) showed a positive distortion bias, and mean over-estimation of the proportion of sequences they repeated correctly (*M* = 0.23, *SD* = 0.15) was significantly different from zero, *t*(58) = 11.83, *P* < .001, one-tailed, *d* = 1.54 (Cohen, 1988).

There was a broad spread of mood symptom scores across the sample as intended (MASQ-S total: *M* = 122.08, *SD* = 31.14, range 72–203; BDI-I: *M* = 9.18, *SD* = 7.77, range 0–35; STAI trait: *M* = 42.41, *SD* = 11.47, range 20–67; STAI state: *M* = 34.98, *SD* = 9.90, range 20–76). The relationships between the various mood disorder symptom measures and distortion are shown in Table 1.

Consistent with Hypothesis 2, as symptom severity increased the extent of the positive distortion bias decreased. This was significant for: MASQ total score, *r* = −.29, *P* = .02, one-tailed; STAI trait, *r* = −.38, *P* < .001, one-tailed; BDI-I total score, *r* = −.23, *P* = .04, one-tailed; and showed a trend towards significance for STAI state, *r* = −.20, *P* = .07, one-tailed. Further, a multiple correlation computed across measures, entering distortion as the independent variable and BDI-I, STAI state, STAI trait and MASQ total scores as the independent variables in a regression analysis, was also significant, *R*(4,54) = .41, *P* = .02, one-tailed.

**Fig. 1:**
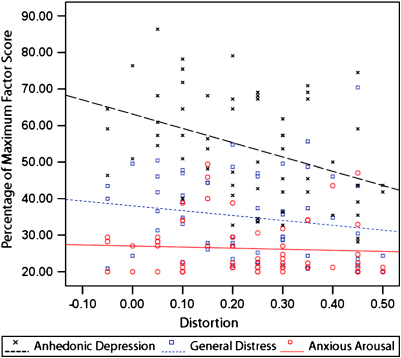
The relationship of distortion with MASQ-S general distress, anxious arousal and anhedonic depression factors (*N* = 59). Note: Scale scores are transformed into percentage of maximum factor score to aid visual comparison. Statistical analyses used untransformed factor scores.

To examine Hypothesis 3, we first explored the relationship of distortion to the MASQ-S AD subscale compared with the association between distortion and the other MASQ-S factors (see Table 1 and Fig. 1). Consistent with predictions, there was a moderate and significant negative correlation between distortion and AD, *r* = −.41, *P* < .001, one-tailed, a small and non-significant correlation between distortion and GD, *r* = −.18, *P* = .19, and a trivial and non-significant correlation between distortion and AA, *r* = −.06, *P* = .66. As predicted, the magnitude of distortion correlations was significantly greater for AD than for the remaining MASQ items (pooling AA and GD), *t*(56) = 2.94, *P* = .002, one-tailed, *d* = .79. Moreover, the distortion-AD correlation was large and significant even after partialling out the remaining MASQ items, *r_p_* = −.46, *P* < .001, one-tailed.

Next, we explored the relationship of distortion with the anhedonia items extracted from the STAI-T, STAI-S and BDI-I. STAI-T items assessing “depression” symptoms (Bieling et al., 1998) showed a moderate and significant negative correlation with distortion, *r* = −.42, *P* = .001, one-tailed. While the remaining STAI-Trait “anxiety” items also showed a moderate significant correlation with distortion*, r* = −.26*, P* = .05, the magnitude of this correlation was significantly smaller than for the “depression” items, *t*(56) = 1.91, *P* = .03, one-tailed, *d* = .51. The correlation of distortion with the “depression” factor remained moderate and significant when partialling out the effects of the remaining “anxiety” items, *r_p_* = −.37, *P* < . 01, one-tailed, whereas notably the “anxiety” items correlation with distortion was small and non-significant when partialling out the effects of “depression” items, *r_p_* = .12, *P* = .36.

STAI-S items measuring reduced “positive affect” (Mathews & Mackintosh, 2000), were moderately and significantly associated with distortion, *r* = −.30, *P* = .01, one-tailed, whereas the remaining STAI-S items showed a small and non-significant correlation, *r* = −.14, *P* = .27, with the magnitude of these correlations again differing significantly, *t*(56) = 2.01, *P* = .024, one-tailed, *d* = .54. Reduced “positive affect” was still moderately and significantly related to distortion when partialling out the remaining STAI-state items, *r_p_* = −.31, *P* = .01, one-tailed.

Finally, BDI-I items measuring “psychomotor anhedonia” (Dunn et al., 2002) showed a significant and moderate correlation with distortion, *r* = −.34, *P* < .001, one-tailed, whereas the correlation with the remaining BDI-I items was small and non-significant, *r* = −.17, *P* = .20, with the magnitude of these correlations again significantly differing, *t*(56) = 2.09, *P* = .02, one-tailed, *d* = .56. The relationship between BDI-I anhedonia items and distortion remained moderate and significant even when partialling out the remaining BDI-I items, *r_p_* = −.33, *P* = .01, one-tailed.

An identical pattern of results emerged when all analyses were repeated co-varying for gender, medication status, age and NART estimated IQ, suggesting that the findings can in no significant way be accounted for by demographic confounds.

## Discussion

The present study examined whether a specific cognitive processing profile for depression could be identified by adopting a dimensional rather than categorical model of the construct and by focusing on variations in positive, rather than negative, information processing bias. Participants with mixed mood disorder symptoms completed a self-judgment task, which had previously revealed that dysphoric and depressed individuals show an attenuated, but still positive, distortion bias relative to healthy participants (B. D. Dunn et al., 2007).

In support of our first hypothesis, participants reliably showed a positive distortion bias on the task, with 92% estimating that they had performed better than they actually had. Validating our second hypothesis, the extent of this positive distortion bias decreased as mood disorder symptom severity increased. This relationship was significant in a multiple correlation analysis across all three mood measures with a medium effect size (Cohen, 1998). There was also a significant negative relationship between distortion and MASQ-S, BDI-I and STAI-T total scores analysed individually, albeit with small effect sizes (Cohen, 1988), and a trend towards significance for STAI-S scores. That the magnitude of the correlation was higher for the three measures assessing symptoms over the past week than for state anxiety suggests distortion is influenced more by background mood than by current emotional state.

Consistent with our third and central hypothesis, the relationship between positive distortion bias on the self-judgment task and mood disorder symptoms on the MASQ-S was found to be predominantly governed by anhedonic symptoms specific to depression. This was evidenced by the fact that this MASQ-S AD-distortion negative correlation: was moderate and significant; was significantly greater than the non-significant correlations between distortion and other MASQ-S items; and was large and significant after partialling out scores on these other items. Validating these results, the same picture emerged, albeit to a less clear extent, when fractionating the BDI-I, STAI-S and STAI-T into anhedonic versus other symptom clusters.

That the MASQ-S findings were clearer than the BDI-I and STAI findings for Hypothesis 3 is unsurprising as this was the only scale that contains substantive numbers of anhedonia items, that was specifically designed to distinguish anhedonic symptoms unique to depression from other features of mood disorders, and for which the factor solution has been subject to extensive psychometric validation.

The present findings have several theoretical implications as to how to best conceptualise depression. That a reduction in a positive distortion bias is uniquely significantly related to the MASQ-S AD factor offers good support for the tripartite model (Clark & Watson, 1991). As far as we are aware this is the first convincing demonstration that MASQ factors uniquely relate to underlying information processing biases, therefore developing construct validity for this framework (though see Larson et al., 2007 for similar work on affective responses). This finding also suggests that viewing depression as a dimensional rather than categorical construct may be a particularly fruitful avenue for further revealing ‘process specificity’ in the disorder.

The demonstration of a reduced positive self-judgment bias in depression is broadly consistent with the wider emotional literature showing that it may be variation in blunted response to positive stimuli rather than elevated response to negative stimuli that is central to depression. Studies reliably demonstrate that depressed individuals differ from control participants in terms of reduced ratings of arousal, valence and happiness when viewing positive material, but do not consistently differ in their subjective ratings of negative material (e.g. Bylsma, Morris & Rottenberg, 2008; B. D. Dunn, Dalgleish, Lawrence, Cusack, & Oglivie, 2004; Rottenberg, Kasch, Gross, & Gotlib, 2002; Sloan, Strauss, Quirk, & Stajovik, 1997). Similarly, depressed individuals find it more difficult to recognise positive than negative facial expressions (i.e. require greater intensity of stimuli before recognition; Joormann & Gotlib, 2006). Finally, it has recently been shown that depressed individuals show reduced reward responsiveness on a probabilistic reward learning task, and that this deficit is to some extent associated with symptoms of anhedonia (Pizzagalli, Iosifescu, Hallett, Ratner, & Fava, 2009).

The current findings, if replicated and extended, also have potential clinical relevance. They suggest that greater attention should be paid to understanding and treating reductions in positive, as well as exaggerations in negative, responses in depression, whether in terms of information processing bias or affective reactions. Dominant theoretical models of depression acknowledge this point to an extent. For example, classic cognitive theory identifies ‘discounting the positives’ as a common thinking error in depression (e.g. Beck, 1995) and the importance of examining positive content in models of specificity has been highlighted (Clark, Beck, & Alford, 1999). Nevertheless, the present findings suggest that an even stronger clinical emphasis on augmenting positivity is required, for example utilising interventions from the positive psychology literature in combination with conventional cognitive therapy approaches (Seligman, Rashid, & Parks, 2006). That depressed individuals show a positive judgment bias, albeit smaller than that of control participants, is also interesting in the context of “reality testing” techniques commonly advocated in cognitive approaches. In some scenarios use of such approaches could further lower mood, removing a potentially protective positive bias (cf. Taylor & Brown, 1988).

Further clarification of the mechanisms underpinning these positive disturbances seems warranted. For example, the present data suggest that the presence of a blunted positive self-judgment bias could plausibly underpin the emergence of anhedonic symptoms. Altered perception of performance by depressed individuals may reduce the degree of reinforcement they receive from activities of daily living. This could lead to behavioural inactivation, including reduced participation in events that have the potential to be positively reinforcing (e.g. Lewinsohn & Graf, 1973), and therefore to a loss of pleasure. Clinically this would suggest that targeting this reduced positive self-judgment bias might facilitate behavioural activation interventions for depression (for a review, see Hopko, Lejuez, Ruggiero, & Eifert, 2003). Future research could usefully examine the validity of this possible mechanism in an ecologically valid setting. Similarly, it will be interesting to explore whether reductions in positive self-judgment bias are related to activity in the behavioural approach system (BAS; see Depue & Iacano, 1989; Pickering and Gray, 1999), given that BAS disturbances have been consistently found in depression (e.g. Kasch, Rottenberg, Arnow, & Gotlib, 2002).

The present study suffers from potential limitations. First, we indexed positive bias solely in terms of self-judgment accuracy and it remains to be demonstrated whether similar conclusions about its unique association with anhedonic symptoms will emerge in other processing domains. Second, participants were selected on the basis of self-report of depression and anxiety symptoms on the MASQ-S and not on symptom counts or severity scores from clinical interviews. Because we were particularly interested in assessing the dimensional factors identified in the tripartite model, a continuous self-report questionnaire approach seemed optimal. Furthermore, in our previous study (B. D. Dunn et al., 2007) we found identical patterns of results in dysphoric participants identified using questionnaires and individuals diagnosed with Major Depressive Disorder identified using structured clinical interviews, and therefore feel this issue is unlikely to have unduly influenced the results.

Third, given that we are arguing that a reduction in positive distortion bias is uniquely associated with depression-specific symptoms, at face value it is surprising that the distortion correlation was numerically greater for STAI-T total score (*r* = −.38) than for the BDI-I (r = −.23) and MASQ (*r* = −.29) total scores. This may reflect the fact that the STAI-T, whilst originally designed as a measure of anxiety, actually includes a greater proportion of anhedonia questions (13 out of 20 items; 65%) than both the MASQ-S (22 out of 62 items; 35%) and the BDI-I (5 out of 21 items; 24%). This suggests that the STAI trait scale may actually be a better measure of anhedonia than it is of anxiety.

Fourth, the present study does not directly contrast dimensional versus categorical models of depression, making it premature to draw strong conclusions about the relative utility of these different conceptual frameworks. It would therefore be interesting to compare within the same sample the capacities of categorical versus dimensional partitioning of depression and anxiety to demonstrate processing specificity.

Finally, stronger evidence for the ability to distinguish anxiety and depression dimensions at the level of cognitive bias would be provided by showing a double dissociation across two cognitive measures. For example, future research could usefully examine whether the anxious-arousal dimension of the MASQ uniquely predicts variation in some other information processing task.

In conclusion, the present study shows that specific cognitive processing biases underlying depression may be more clearly identified by viewing the construct in dimensional rather than categorical terms, and by focusing on variations in the degree of positive, as opposed to negative, bias. The data provide further support for dimensional models of mood disorders such as the tripartite framework (Clark & Watson, 1991) and suggest that examination of the profiles of cognitive processing associated with these dimensions is a promising direction for future research.

## Figures and Tables

**Table 1:** Distortion and mood symptoms correlations (*N* = 59).

		1	2	3	4	5	6	7	8	9	10	11	12	13
Distortion (1)	*r*													
	*P*													
MASQ-S total (2)	*r*	−0.29												
	*P*	_0.01*_												
MASQ-S GD (3)	*r*	−0.18	0.94											
	*P*	_0.19_	_0.00_											
MASQ-S AA (4)	*r*	−0.06	0.60	0.51										
	*P*	_0.66_	_0.00_	_0.00_										
MASQ-S AD (5)	*r*	−0.41	0.93	0.81	0.35									
	*P*	_0.00*_	_0.00_	_0.00_	_0.01_									
STAI-T (6)	*r*	−0.38	0.87	0.80	0.41	0.87								
	*P*	_0.00*_	_0.00_	_0.00_	_0.00_	_0.00_								
STAI-T “anxiety” (7)	*r*	−0.26	0.77	0.71	0.38	0.76	0.90							
	*P*	_0.05_	_0.00_	_0.00_	_0.00_	_0.00_	_0.00_							
STAI-T “depression” (8)	*r*	−0.42	0.85	0.78	0.39	0.86	0.97	0.77						
	*P*	_0.00*_	_0.00_	_0.00_	_0.00_	_0.00_	_0.00_	_0.00_						
STAI-S (9)	*r*	−0.20	0.74	0.71	0.47	0.65	0.69	0.62	0.68					
	*P*	_0.07*_	_0.00_	_0.00_	_0.00_	_0.00_	_0.00_	_0.00_	_0.00_					
STAI-S reduced “positive affect” (10)	*r*	−0.30	0.69	0.63	0.36	0.68	0.73	0.59	0.75	0.90				
	*P*	_0.01*_	_0.00_	_0.00_	_0.00_	_0.00_	_0.00_	_0.00_	_0.00_	_0.00_				
STAI-S “other” (11)	*r*	−0.14	0.71	0.71	0.49	0.60	0.64	0.60	0.60	0.98	0.80			
	*P*	_0.27_	_0.00_	_0.00_	_0.00_	_0.00_	_0.00_	_0.00_	_0.00_	_0.00_	_0.00_			
BDI-I (12)	*r*	−0.23	0.82	0.76	0.39	0.80	0.77	0.74	0.73	0.58	0.56	0.56		
	*P*	_0.04*_	_0.00_	_0.00_	_0.00_	_0.00_	_0.00_	_0.00_	_0.00_	_0.00_	_0.00_	_0.00_		
BDI-I “psychomotor anhedonia” (13)	*r*	−0.34	0.72	0.62	0.37	0.75	0.73	0.73	0.67	0.49	0.49	0.46	0.89	
	*P*	_0.00*_	_0.00_	_0.00_	_0.00_	_0.00_	_0.00_	_0.00_	_0.00_	_0.00_	_0.00_	_0.00_	_0.00_	
BDI-I “other” (14)	*r*	−0.17	0.80	0.78	0.37	0.77	0.75	0.70	0.71	0.58	0.56	0.56	0.98	0.78
	*P*	_0.20_	_0.00_	_0.00_	_0.00_	_0.00_	_0.00_	_0.00_	_0.00_	_0.00_	_0.00_	_0.00_	_0.00_	_0.00_

Distortion = proportion of trials judged as correct minus proportion of trials performed correctly. MASQ-S = Mood and Anxiety Symptom Questionnaire-Short Form. Total score correlations are presented along with correlations for the anhedonia (AD), anxious arousal (AA), and general distress (GD) factors. STAI = Spielberger State Trait Anxiety Inventory. State (-S) and trait (-T) total score correlations are presented along with correlations for trait sub-scale scores for “anxiety” and “depression” items (Bieling et al., 1998) and state sub-scale scores for reduced “positive affect” and “other” items (Mathews & Mackintosh, 2000). BDI-I = Beck Depression Inventory. Total score correlations are presented along with correlations for sub-scales of “psychomotor anhedonia” and “other” symptoms (Dunn et al., 2002). *P values are one-tailed as correlations examine a priori hypotheses.

## Footnotes

1 Recent evidence suggests, however, that anxious arousal is more specifically related to panic than to anxiety generally and that anhedonia symptoms may be associated with social phobia as well as depression (Brown, Chorpita, & Barlow, 1998; Mineka et al., 1998).

2 It could be argued that the current experimental rationale is somewhat tautological in nature in that it simply aims to demonstrate that subjective symptoms of blunted positivity are correlated with objective measures of blunted positive information processing. In our view however it is theoretically and clinically useful to distinguish between accurate description of *phenomenology* (in this case anhedonic symptoms of depression) and detailed identification of the information processing *mechanisms* that might underpin this (in this case, reduced positive self-judgment bias). By identifying both phenomenology and mechanism it is possible to develop a more helpful model of disorder maintenance and to devise effective interventions. For example, a large body of work has established that negative judgment bias mechanisms are related to negative affect symptoms across a range of mood disorders (for a review see Mineka et al., 2003) and this has formed the basis of cognitive interventions for these conditions.

3 To ensure a good spread of depression and anxiety symptoms in the present sample, we requested two lists of volunteers from the participant panel, one comprising individuals with depression and anxiety scores in the non-clinical range and with no history of mood disorders and one comprising individuals with depression and anxiety scores above non-clinical ranges. Thirty individuals were recruited from each list. Given this recruitment method, it is possible that a bimodal distribution might have been obtained. However, exploratory data analysis revealed that MASQ-S total scores were normally distributed across the sample, kurtosis = −.64, skewness = .33, Kolmogorov–Smirnov test of normality (59) = .11, *P* > .05. Further, participants recruited from either list did not significantly differ in terms of age, NART estimated intelligence, ts < 1, or gender ratios, *χ*^2^ < 1, suggesting that those with more extreme scores did not represent a unique sample in terms of other characteristics (cf. Coyne, 1994).

4 The BDI-I rather than the BDI-II was selected as we were aware of a factor analytic study that had a found an anhedonia specific dimension for the BDI-I (Dunn et al., 2002) but do not know of any studies that have found a clear anhedonia factor for the BDI-II. Further, our subject panel stores BDI-I rather than BDI-II data for historical reasons. These BDI-I results need to be interpreted cautiously however, as the Dunn et al. (2002) factor analysis was conducted on a relatively small sample and generated a somewhat unclean factor solution. Further, the size of the correlation with the MASQ-S AD factor was comparable for the BDI-I anhedonia and “other” items in the present sample (see Table 1), suggesting that it is not a particularly sensitive measure of the anhedonic symptoms of depression. It will be important to replicate these results using the BDI-II and other measures of depression. In contrast, the anhedonia items extracted from the STAI-T and STAI-S had larger correlations with MASQ-S AD factor than did the remaining “other” items on both scales, suggesting they are adequate measures of anhedonia.
